# Bioaugmented osteosynthesis: precise monitoring and intervention of the bone healing microenvironment

**DOI:** 10.1038/s41413-025-00466-5

**Published:** 2025-11-25

**Authors:** Gaoxiang Xu, Junyang Chen, Zhikang Xiao, Jianzhong Xu, Licheng Zhang, Peifu Tang

**Affiliations:** 1 Department of Orthopedics, the Fourth Medical Center, Chinese PLA General Hospital, Beijing, China; 2 Department of Orthopedics, The First Affiliated Hospital of Zhengzhou University Zhengzhou, Zhengzhou, China; 3 School of Life Sciences, Zhengzhou University, Zhengzhou, China; 4 National Clinical Research Center for Orthopedics, Sports Medicine & Rehabilitation, Beijing, China

**Keywords:** Pathogenesis, Calcium and phosphate metabolic disorders

## Abstract

Bone healing is integral to orthopedic research, focusing on the restoration of bone function through a complex interplay of inflammatory responses, soft callus formation, hard callus development, and the final remodeling phase. While the natural progression of bone healing is a finely tuned process, it can be disrupted by inflammatory dysregulation, ranging from chronic inflammation to acute inflammatory anomalies, and by the depletion of essential repair substances under both chronic and acute conditions. Current strategies to enhance bone healing employ a multifaceted approach, including biochemical modulation of the local microenvironment through essential nutrient supplementation (e.g., calcium and vitamin D), biomechanical optimization via improved internal fixation stability, and advanced regenerative techniques incorporating bioactive factors, stem cell therapies, and functional biomaterials. Despite these efforts, challenges persist in the precise characterization of the local microenvironment and the precise control of in vivo bioactive molecule delivery. This review comprehensively summarizes the current research progress in bone healing, providing significant reference for understanding the mechanisms of bone healing and for guiding further research. It is expected to lay the theoretical foundation for the development of more effective therapeutic strategies for bone healing.

## Introduction

Investigations into bone healing are of paramount importance in contemporary medicine, as they pertain to the restoration of function and the enhancement of quality of life for patients with skeletal injuries. The occurrence of skeletal trauma, such as fractures, is a frequent event in clinical practice, and the process of bone healing is an intricate biological cascade involving multiple cell types, numerous regulatory factors, and a specific sequence of events. The healing of a bone initiates with the inflammatory response phase, where inflammatory cells congregate at the fracture site and release mediators that trigger subsequent reparative actions.^1–3^ This phase is followed by the formation of a cartilaginous callus, the formation of a hard callus, and finally, the remodeling phase. During the cartilage callus phase, mesenchymal stem cells (MSCs) differentiate into chondrocytes, constructing cartilage-like tissues^4^; in the hard callus phase, these cartilaginous tissues are incrementally replaced by hard bone tissues with ongoing new bone formation^5,6^; and in the remodeling phase, the newly formed bone tissues are refined for structural and mechanical optimization to accommodate the body’s functional demands.

Nevertheless, the bone healing process is vulnerable to various influences. Dysregulation of inflammation is a pivotal factor; acute inflammatory deviations, such as excessive, insufficient, or suppressed inflammation, impede the healing process.^7–9^ Additionally, the depletion of reparative substances, whether chronic or acute, diminishes the body’s reparative capabilities, particularly in the context of inadequate cell counts and mineral deficiencies.^8^ Furthermore, skeletal muscle weakness reduces osteogenic capacity.^10–12^ Recent advancements in biomaterials science have introduced new opportunities and challenges in bone healing research. Biomaterials are anticipated to enhance the efficacy of reparative substances and improve bone healing by modulating the bone healing microenvironment, including providing sites for cell adhesion and delivering bioactive factors.^13^ Concurrently, biosensors leverage accessible clinical specimens to enable timely, precise monitoring of subtle microenvironmental shifts. This capability facilitates immediate therapeutic adjustment and early intervention to minimize complications.

The purpose of this review is to delve into the underlying mechanisms of bone healing, scrutinize the influencing factors, and systematically compile the related research findings to offer a comprehensive reference for future studies, thereby fostering the evolution of orthopedic medicine and elevating the effectiveness of bone healing.

## Bone healing process

**Fig. 1:**
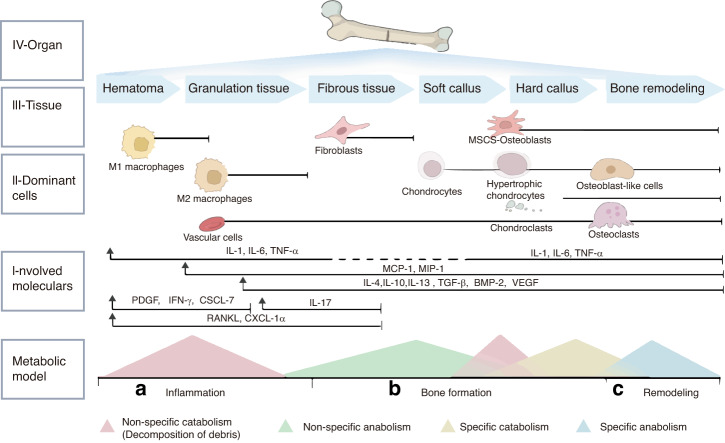
The process of bone repair is a dynamic and ongoing metabolic cycle, characterized by distinct phases where various cell types and cytokines assume pivotal roles. These phases include: **a** the inflammatory response, **b** the phase of osteogenesis, and **c** the phase of bone remodeling. Each stage is marked by the predominant activity of specific cellular and molecular mediators

Bone, as a continuous regenerative tissue, consists of mineralized extracellular matrix and several types of cells hosted in the tissue. The mineralized extracellular matrix constituted by organic matter and inorganic matter.^14^ Bone cells include MSCs, osteoblasts and osteocytes forming bone, osteoclasts resorbing bone.^15^ Under normal physiological conditions, bone tissue healing is characterized by two principal processes: primary, also known as direct healing, and secondary, or indirect healing.^16^ Direct fracture healing is occurred as the anatomical reduction is accomplished and strong fixation is achieved, and the Haversian system also heals directly. Clinically, most fractures heal indirectly, which is a combination of two types of bone formation: intramembranous ossification and endochondral ossification. During indirect fracture healing, intramembranous ossification occurs faster than endochondral ossification, and intramembranous ossification mainly involves the periosteum. Therefore, the periosteum must be specially protected. Herein, we focus on the more common indirect bone healing processes, encompassing the inflammatory, Cartilage scab formation, hard bone scab formation, and plastic phases (Fig. 1).

### Inflammatory phase

After the initial fracture, the local vascular network and tissues are destructed, followed by the formation of hematomas from cells in the peripheral and intramedullary hematopoietic compartments, which induces the emergence of an acute inflammatory phase.^1–3^ Throughout this process, plasma coagulation and platelets are subjected to the extracellular milieu, collectively furnishing a fibrin scaffold that serves as the principal provisional matrix, alongside a dense constellation of angiogenic growth factors exhibiting robust pro-angiogenic capabilities.^1,17^ At the site of the hematoma, the matrix, along with dead cells and debris, initially attracts polymorphonuclear neutrophils. These neutrophils then release pro-inflammatory cytokines and chemotactic agents, such as interleukin 6 (IL-6) and C-C motif chemokine 2 (CCL2), which signal for a subsequent influx of inflammatory cells including monocytes, macrophages, and lymphocytes.^18,19^ Within this inflammatory context, macrophages differentiate into a pro-inflammatory M1 phenotype.^20^ Within the inflammatory environment at the hematoma site, immune cells migrate to clear out the provisional fibrin matrix and eliminate necrotic cells. Concurrently, osteoclasts are tasked with the resorption of dead bone fragments. Following this clearance, macrophages transition towards an anti-inflammatory M2 phenotype, secreting a variety of mediators that include stromal derived factor-1α (SDF-1α), IL1β, IL-6, tumor necrosis factor-α (TNF-α), CCL2, bone morphogenetic proteins (BMPs), and fibroblast growth factors (FGFs). This secretion initiative is crucial for the recruitment of progenitor cells to the area.^19,21^ In the week that follows, the hematoma and its accompanying acute inflammation are resolved, with the hematoma being supplanted by granulation tissue. This new tissue is rich in multiplying progenitor cells and a burgeoning neovascular network, all embedded within an extracellular matrix that is free of surrounding tissue.

The site of injury transitions into a phase dedicated to fracture healing and the restoration of skeletal tissue, encompassing two principal stages: the initial anabolic and subsequent catabolic phases. The anabolic phase is distinguished by a surge in tissue volume due to the de novo recruitment and differentiation of stem cells into bone and vascular structures, primarily encompassing the cartilaginous and sclerotic callus formation stages.

### Stage of fibrocartilaginous Callus formation

The synergistic action of growth factors, such as Transforming Growth Factor-beta 2 and 3 (TGF-β2 and -β3), Platelet-Derived Growth Factor (PDGF), Fibroblast Growth Factor-1 (FGF-1), and Insulin-Like Growth Factor (IGF), stimulates the proliferation and differentiation of MSCs into fibroblasts, osteoblasts, and chondrocytes.^1^ This process orchestrates the synthesis of the cartilage matrix, leading to a comprehensive replacement of fibrin and granulation tissue with cartilaginous structures.^19^ In instances where chondrogenesis is lacking, fibroblasts undergo differentiation into fibrous tissue, thereby filling in the affected region. SOX9, together with the transcriptional cofactors SOX5 and SOX6, regulates the expression of collagen II (or X collagen in hypertrophic chondrocytes) and aggregated proteoglycans in chondrocytes, conferring characteristic biophysical properties on cartilage and stabilizing defects. Meanwhile, the vascular endothelial cells are stimulated by stimuli including VEGF, FGF-1, BMP and TGF-β to achieve vascular endothelial cell invasion, angiogenesis and capillary growth in the soft healing tissue. Proximal to the fracture line site, cartilage healing tissue was formed.

### Stage of hard callus formation

During the third stage of bone healing, cartilage size gradually increases and calcification begins. In areas where mechanical stability is low, CXCL12 is secreted by the periosteum under the control of HIF1α, inducing MSCs mobilization from the periosteum and bone marrow near the fracture site, where they adhere to the fracture area. These MSCs commit to an osteoblastic lineage, leading to the direct formation of woven bone. This process is induced by E11 and Cx43. Upon achieving mechanical stability, the central soft callus undergoes hypertrophy and subsequent mineralization by the initially formed bone matrix.^4^ During chondrocyte hypertrophy, there is a downregulation of SOX9 and an upregulation of Runx2 and β-linker protein expression. This is succeeded by the induction of alkaline phosphatase, osterix, osteopontin, and osteocalcin, which facilitate the calcification of the cartilaginous matrix. Additionally, chondrocytes express components of the Wnt signaling pathway, including Dishevelled and β-catenin, which are instrumental in their transition to an osteoblast/osteoclast phenotype, leading to the formation of woven bone.^22^ Throughout the formation of a hard bone callus, cellular recruitment and differentiation take place within the encircling myosin sheath, resulting in the generation of new blood vessels that nourish the emerging bone. Osteoclasts are mobilized by macrophage colony-stimulating factor (M-CSF) and receptor activator of nuclear factor-κB ligand (RANKL) to resorb the initial woven bone, thereby facilitating the deposition of new lamellar bone.^5,6^ TNF-α stimulates osteoclast function, which resorbs mineralized cartilage. However, Gerstenfeld et al. demonstrated that TNF-α signaling is essential for initiating endochondral ossification during fracture healing.^23^ Additionally, it can synergize with IL-1β to promote matrix mineralization of MSC.^24,25^ During this process, matrix metalloproteinases, specifically MMP13 and MMP9, break down cartilage in endochondral ossification by degrading type II collagen into gelatin.^6,22,24–26^

### Bone remodeling

During this stage, the superior lamellar bone steadily replaces the woven bone, while the overall size of the healing tissue decreases and normal hematopoietic and trabecular structures are restored. Osteoblasts coordinate the remodeling process in the cellular compartment, with osteoclasts resorbing hard healing tissue and osteoblasts constructing lamellar bone.^6,22,24–26^ The regulation of bone healing is essential and is achieved through MSC/macrophage crosstalk.^27^ M1-polarized macrophages, which are proinflammatory, promote the mineralization of MSCs partly through the production of oncostatin M. On the flip side, MSCs facilitate mineralization in macrophages through thrombospondin (TSG). Additionally, MSCs stimulate a phenotypic switch in macrophages from the pro-inflammatory M1 state to the anti-inflammatory M2 state via TSG-6 and prostaglandin E2 (PGE2), consequently diminishing the secretion of proinflammatory cytokines.^28,29^ The remaining osteoclasts are directed towards apoptosis through the activation of their c-FMS receptors. In the context of human bone, this process promotes the restoration of the Haversian system. This system consists of a central vascular canal that supplies a network of branching tubules, with osteoblasts and osteoclasts as central components. Matrix extracellular phosphoglycoprotein (MEPE) and osteocalcin are important regulatory molecules for this process.^30–32^

**Fig. 2:**
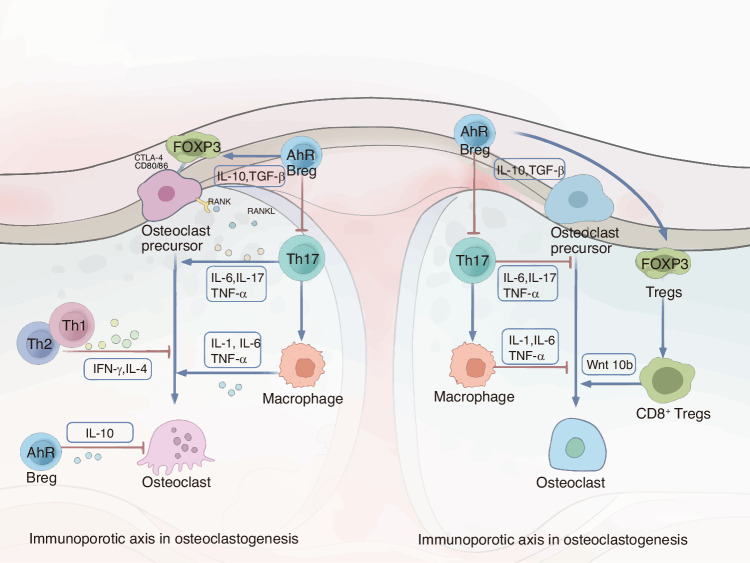
Delineates the immunomodulation of bone remodeling and potential therapeutic targets: osteoclast differentiation is a complex process influenced by various immune cells. Regulatory B (Breg), regulatory T (Treg), Th1, and Th2 cells suppress osteoclast maturation from precursors through the production of anti-inflammatory cytokines like IL-10, TGF-β, IFN-γ, and IL-4, thereby inhibiting osteoclastogenesis. Conversely, Th17 cells promote osteoclastogenesis through the secretion of inflammatory cytokines, either directly or indirectly. Tregs promote osteoblastogenesis, particularly via CD8^+^ Tregs, while Th17 cells hinder this process, either by IL-17 production or by amplifying macrophage-mediated reduction in osteoblast differentiation

During the whole fracture healing phase, moderate inflammation, adequate cell and nutrients are the key to the normal bone healing. Simultaneously, the immune system contributes to the ongoing process (Fig. 2).

## Disturbed bone healing microenvironment

A systematic overview of the bone healing process reveals the necessity of two key factors for the orderly progression of the bone healing cascade: an appropriate inflammatory response, including a balance of pro- and anti-inflammatory factors, and an adequate supply of reparative substances, including cells and minerals. It is unfortunate that the disruption of these two elements-namely, an abnormal inflammatory response and a lack of reparative substances-can occur in different states of the body, which in turn disrupts the overall process of bone healing.

### Dysregulated inflammation

**Fig. 3:**
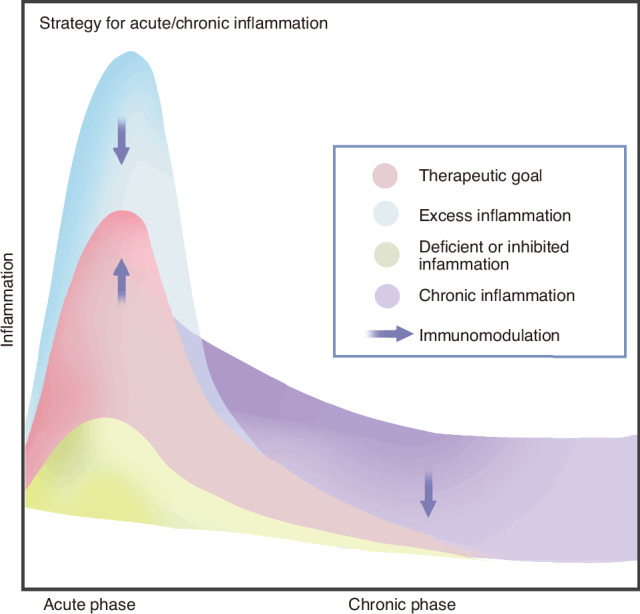
Strategies for modulating immunity to facilitate bone repair can be differentiated for scenarios of acute and chronic inflammation. The ideal inflammatory response for bone regeneration begins with a precise and temporary phase of acute inflammation, which is then followed by its resolution. Both an overabundance and a lack or suppression of acute inflammation, as well as the presence of chronic inflammation, can impede the restoration of bone defects

As previously discussed, inflammation is a crucial initial step in bone healing (Fig. 3). Consequently, the absence and suppression of acute inflammation can impair osteogenesis. For instance, it is well-documented that medications such as nonsteroidal anti-inflammatory drugs (NSAIDs), corticosteroids, and chemotherapeutic agents can elevate the risk of nonunion in bones.^33^ Moreover, excessive acute inflammation resulting from severe injuries like multiple traumas and open fractures also increases the risk of compromised bone healing.^8^ Additionally, the dyshomeostasis caused by chronic inflammation is detrimental to the oweisteogenic process.^8^

#### Chronic inflammation and bone healing

Chronic inflammation disrupts bone healing through multiple mechanisms centered on the synergistic effects of dysregulation of inflammatory mediators and depletion of repair substances. In chronic inflammatory environments, persistent activation of TNF-α and NF-κB signaling pathways not only directly promotes osteoclast differentiation and bone resorption,^34^ but also impairs bone regeneration by inhibiting the osteogenic potential of bone marrow mesenchymal stem cells (BM-MSCs).^35^ Senescence, a typical trigger of chronic inflammation, significantly reduces angiogenesis and local perfusion, leading to a decrease in the number of cells in the fracture region and an inadequate nutrient supply.^36^ Additionally, the decline in bone-promoting biological factors secreted by skeletal muscles, induced by senescence and other chronic diseases, results in a reduction in bone regeneration.^10^ Although the fracture healing program at the molecular level is not directly affected by aging, bone tissue volume, mineral content, and mechanical properties are significantly reduced in aging individuals.^37^ In addition, the quality and quantity of periosteal and bone marrow-derived stem cells decline with aging, and osteogenic progenitor cells have a reduced ability to differentiate and delay the initial fracture response.^38^ For example, reduced regulatory T cells (Treg) in older individuals lead to uncontrolled inflammatory responses, shortened macrophage lifespan, exacerbated oxidative damage,^39^ and insensitivity to GM-CSF stimulation,^40,41^ which further impairs bone healing. Systemic inflammation in diabetic patients disrupts chondrocyte function through elevated ROS and TNF-α,^42^ accelerates cartilage degradation and increases bone resorption, significantly increasing the risk of delayed fracture healing. Inflammatory bowel disease (IBD) patients suffer from inadequate absorption of calcium, vitamin D and vitamin K due to intestinal barrier dysfunction,^43^ exacerbating osteoporosis and impaired bone regeneration.^44^ Animal studies have shown that IBD promotes bone loss by disrupting the microbial-gut-bone axis,^45^ whereas clinical studies have confirmed a significantly higher incidence of postoperative complications after hip arthroplasty in patients with IBD.^46^ Notably, sustained activation of M1 macrophages and overproduction of cytokines such as IL-1, IL-6, and TNF-α not only directly enhanced osteoclastogenic activity, but also promoted osteoclast activity and bone resorption through elevated levels of TRAP and IFN-γ,^47–49^ as well as inhibited osteogenesis-related gene expression impede the formation of bone scab, creating a vicious cycle of “inflammation-repair imbalance”. Aging also leads to increased apoptosis of osteoblasts and the development of large cavities in the trabeculae, which further weaken the mechanical stability of the bone structure.^40,41^

#### Excess of inflammation and bone healing

Excessive activation of acute inflammation severely disrupts bone healing through cytokine storms and immune microenvironment dysregulation. Rapid release of TNFα and NF-κB post-trauma not only enhances osteoclast activity^34^ but also directly inhibits osteoblast differentiation by suppressing the Wnt/β-catenin pathway. Systemic inflammatory response syndrome (SIRS) induced by polytrauma interferes with early regenerative signals at the fracture site, while concomitant skeletal muscle injury causes acute depletion of osteogenic progenitor cells and myokines,^50^ further compromising bone repair capacity. Infection sustains elevated levels of pro-inflammatory factors that activate osteoclast precursors^51,52^ and systemically deplete critical repair substrates, including collagen, calcium, phosphorus, vitamin D, and vitamin C,^53^ leading to delayed or non-union of fractures. Clinical data indicate that polytrauma patients exhibit 30%–50% prolonged fracture healing times and 2-3-fold increased nonunion rates compared to isolated fracture cases.^33,54,55^ Additionally, reduced CD4^+^/CD8^+^ T cell ratios post-trauma^56^ and persistent polarization of M1 macrophages^57^ exacerbate local inflammatory imbalance, inhibiting osteogenic differentiation of BM-MSCs. Mechanical instability aggravates this vicious cycle: excessive interfragmentary motion induces capillary rupture and platelet aggregation,^58^ releasing chemokines such as CCL2 and IL-17A,^59,60^ which recruit neutrophils and M1 macrophages,^61^ perpetuating the inflammatory microenvironment. Notably, while skeletal muscle serves as a “secondary periosteum” by supplying osteogenic cells and angiogenic factors during bone repair,^50^ severe muscle injury depletes these resources, impairing callus vascularization and mineralization. Animal studies further demonstrate that polytrauma models with concomitant muscle injury exhibit suppressed osteogenic gene expression and elevated TRAP activity in callus tissues,^50^ highlighting the synergistic detrimental effects of inflammation and repair substance depletion. Thus, excessive inflammation not only directly disrupts repair processes^43,62^ but also indirectly exacerbates bone healing impairment through resource exhaustion.

#### Deficiency and inhibition of acute inflammation

Insufficient or excessive suppression of acute inflammation negatively impacts bone healing, underscoring the biphasic regulatory nature of inflammatory responses. Immune-deficient models, such as NOD/SCID-IL2Rγcnull mice, demonstrate that while early callus formation remains unaffected, delayed endochondral ossification occurs with persistent cartilage remnants and reduced mineralization in later stages.^63^ This highlights the critical role of monocytes, dendritic cells, and T cells in terminal bone remodeling. TNF-α exhibits a biphasic release pattern post-fracture: an initial peak drives inflammatory cell recruitment and chondrogenesis, whereas TNF-α receptor-deficient mice display excessive cartilage accumulation and delayed ossification.^23,64^ Aging-associated defects, such as reduced macrophage responsiveness to GM-CSF and increased osteoblast apoptosis,^40,41^ exacerbate inflammation-resource imbalances, further impairing bone repair. IL-6 facilitates osteoclast precursor fusion during callus remodeling, and its knockout mice exhibit diminished osteoclastogenesis but comparable late-stage callus strength,^65^ indicating stage-specific roles. Deficiencies in chemokines like CCL2 impair macrophage and BM-MSC infiltration, delaying vascularization and callus maturation.^66^ PGE2, synthesized via COX-2, is essential for osteoblastogenesis, as evidenced by impaired healing in COX-2-deficient mice.^67^ Clinical studies confirm that NSAIDs inhibit the COX-2-PGE2 pathway, prolonging fracture healing by 20%-40% and increasing nonunion risk by 1.5–2-fold.^68^ Additionally, acute trauma-induced systemic depletion of collagen, calcium, and vitamin D/C^53,69^ synergizes with anti-inflammatory therapies to exacerbate repair failure. IL-17A, secreted by γδ T cells, stimulates BM-MSC proliferation and osteoblastic differentiation; its deficiency results in reduced callus mineralization and mechanical strength.^70^ Animal models of severe muscle injury combined with immune suppression reveal prolonged M1 macrophage dominance and CD4^+^/CD8^+^ T cell ratio imbalance,^50,57^ further linking inflammation deficiency to impaired osteogenesis. These findings collectively demonstrate that balanced acute inflammation is indispensable for initiating repair, whereas pharmacological interventions or congenital immune deficiencies disrupt the inflammation-repair equilibrium, ultimately delaying or aborting bone healing.

## Bone healing

### Microenvironmental regulation of fracture healing

Calcium and vitamin D play crucial roles in bone health and fracture healing. Calcium is a key structural component of bone, while vitamin D maintains calcium balance, improves muscle function, and prevents deficiencies.^71^ Animal studies demonstrate that vitamin D enhances fracture healing, with 1,25(OH)_2_D_3_ present in healing tissues regulating the process.^71,72^ Active vitamin D metabolites improve bone mechanical properties by increasing newly formed bone volume, callus density, and trabecular count through osteoblast differentiation and extracellular matrix mineralization.^73,74^ Additionally, they boost enzymatic collagen cross-linking, enhancing the ultimate load capacity of healing tissues.^75^ A randomized controlled trial in 30 female patients with proximal humerus fractures showed that calcium and vitamin D supplementation significantly increased bone mineral density at the fracture site.^76^ Bisphosphonates, commonly used to treat osteoporosis, reduce fracture risk, improve bone mineral density, and normalize elevated bone turnover markers. Their chemical structure targets bone, where they inhibit osteoclast activity, slowing bone remodeling.^77^ Animal studies reveal bisphosphonates accumulate at fracture sites, increasing healing tissue volume, trabecular bone, and bone mineral content.^75^ Clinical trials confirm higher bone mineral density at fracture sites with bisphosphonate treatment.^78^ Denosumab, a monoclonal antibody targeting RANKL, inhibits osteoclast formation and function, reducing bone resorption.^79^ Preclinical studies show denosumab does not impair fracture healing; instead, it enhances bone strength at the healing site compared to alendronate and control groups.^80^ The FREEDOM Trial involving 667 postmenopausal women found no delay in fracture healing or complications with denosumab use.^81^ Parathyroid hormone (PTH), particularly PTH 1–34 and PTH 1–84, regulates calcium and phosphate metabolism, promoting osteoblast survival and proliferation.^82^ Animal studies using recombinant human PTH show larger, denser healing tissues with improved biomechanical properties.^83^ Teriparatide (PTH 1–34) accelerates endochondral ossification by enhancing chondrocyte recruitment,^84^ with early post-fracture administration yielding optimal results.^85^ A randomized trial in postmenopausal women with distal radius fractures found teriparatide (20 μg/day) reduced healing time from 9.1 to 7.4 weeks, though higher doses showed no additional benefit.^86^ Post hoc analysis confirmed dose-dependent improvements in early healing tissue formation.^87^

### Acceleration of fracture healing

#### Bioactive factors

Growth factors play pivotal roles in bone healing through their ability to regulate cellular proliferation, differentiation, and angiogenesis. Platelet-derived growth factor (PDGF), existing in five dimeric forms (AA, AB, BB, CC, DD), is primarily secreted by platelet α-granules and stimulates chondrocyte and osteoblast proliferation via PI3K and MAPK signaling pathways.^88^ Among its isoforms, PDGF-BB demonstrates the most potent osteogenic effects, earning FDA approval for clinical applications in fracture healing and bone grafting.^89^ Clinical studies show recombinant human PDGF-BB (rhPDGF-BB) achieves comparable fusion rates to autologous bone grafts in foot/ankle procedures while avoiding donor site morbidity.^90^ However, genetic variants in PDGF may predispose patients to fracture nonunion.^91^ Transforming growth factor-beta (TGF-β) serves as a key regulator of bone remodeling through its concentration-dependent effects on mesenchymal cells. At physiological concentrations, TGF-β promotes osteoblast differentiation via Smad pathway activation and stimulates extracellular matrix production by enhancing collagen and fibronectin synthesis.^92^ This regulatory function positions TGF-β as a critical mediator in the coupling of bone formation and resorption. Vascular endothelial growth factor (VEGF) represents another essential mediator, with its isoforms (VEGF-A, -B, -C, -D, PGF1/2) governing angiogenesis and vascular permeability.^93^ VEGF-A demonstrates particular importance in bone repair by inducing MSC osteogenic differentiation and promoting endothelial cell recruitment.^94^ The establishment of robust vascular networks through VEGF signaling enhances nutrient delivery and waste removal during bone regeneration.^95^ However, therapeutic application requires precise dosing due to VEGF’s narrow therapeutic window-excessive doses cause vascular malformations, while its short half-life (4–24 h) necessitates controlled delivery systems.^96^ Bone morphogenetic proteins (BMPs), particularly BMP2 and BMP7, promote osteogenesis via serine/threonine kinase receptors.^97^ Recombinant human BMPs (rhBMP2 and rhBMP7) are FDA-approved for orthopedic conditions, including open fracture and nonunion.^98^ Studies report successful healing in tibial pseudoarthrosis or persistent tibial nonunion in children and adolescents (9/10 cases)^99^ and refractory upper extremity nonunion (40/42 patients).^100^ However, high BMP-2 doses can cause osteolysis, inflammation, and pain.^101^

#### Stem cells and cell derivatives

**Fig. 4:**
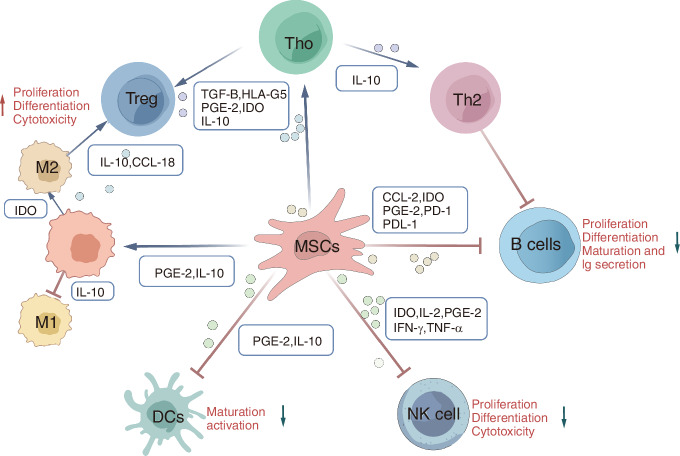
Illustrates the impact of MSC-mediated immunomodulation on immune cell activity. MSCs primarily modulate immune responses through direct interactions with both innate and adaptive immune cells and via their paracrine secretions, which include a diverse array of bioactive molecules. Predominantly, MSCs have an immunosuppressive effect (depicted by red inhibitory symbols), but they also stimulate the development of Treg cells, the differentiation of Th2 cells, and the polarization of M2 macrophages (indicated by green arrows). This immunomodulatory process involves multiple cytokines, chemokines, signaling molecules, and growth factors, which help to preserve immune homeostasis by regulating overactive or insufficient immune responses. Key factors in this mechanism include PGE2, IDO, IFN-γ, TNF-α, TGF-β, various interleukins (IL), the PD-1/PD-L1 axis, and chemokine receptors and ligands such as CCR6, CCL-2, and CCL-18

Bone marrow-derived stem cells (BMSCs) represent the most extensively studied adult mesenchymal stem cells (MSCs) due to their accessibility, proliferative capacity, and regenerative potential (Fig. 4).^102^ Previous studies confirm that allogeneic BMSCs effectively substitute autologous bone grafts in treating osseous defects.^103^ Clinical trials demonstrate their safety, with Kim et al. reporting accelerated fracture healing in 64 patients after local injection of osteogenically differentiated autologous BMSCs (3.0 × 10^7^ cells/mL), without complications.^104^ Similarly, Marcacci et al. achieved complete defect healing within 5–7 months using 2.0 × 10^7^ MSCs/mL, with sustained outcomes over 6–7 years.^105^ Adipose-derived stem cells (ADSCs) require combinatorial strategies to enhance osteogenesis. While direct implantation fails in critical-sized defects,^106^ Di Bella et al. successfully reconstructed cranial defects using ADSCs seeded on fibronectin-coated scaffolds.^107^ Phenylephrine treatment and noggin inhibition further augmented ADSC osteogenesis.^108^ Clinically, ADSCs combined with β-tricalcium phosphate (β-TCP) and BMP-2 restored maxillary defects, while ADSC-cancellous bone composites improved fibular continuity.^109^ Extracellular vesicles (EVs) derived from MSCs modulate bone regeneration via multifaceted mechanisms. BMSC-EVs can enter osteoblasts and deliver osteogenic miRNAs through endocytosis, thereby regulating osteogenic gene expression and promoting bone healing.^110^ ADSC-EVs enhance osteoblast function via Wnt/β-catenin signaling, preserving 82% trabecular volume in osteoporosis models.^111^ Engineered EVs, such as miR-21-5p-enriched or TGF-β1-modified EVs, further optimize repair by reducing apoptosis or inflammation.^112^ Based on the concept of “muscle enhancement and bone strengthening”, Our team promotes the secretion of EVs rich in miR-873-3p by human skeletal muscle myoblasts through the inhibition of histone deacetylase, thereby promoting the osteogenic differentiation of BMSCs and effectively enhancing bone formation.^10^ Biomaterial-loaded EVs improve targeting, with β-TCP scaffolds increasing cell recruitment by 2.7-fold^113^ and decalcified bone matrices boosting angiogenesis by 58%.^114^ However, clinical translation faces challenges like batch heterogeneity and short in vivo half-life.^115^ Bone marrow aspirate (BMA), an FDA-accepted autologous therapy, contains MSCs and osteoprogenitors that directly promote healing.^116^ Hernigou et al. reported 87.4% fracture union rates in Gustilo-Anderson open fractures after BMA injection.^117^ Meta-analyses confirm BMA’s efficacy, particularly when combined with autologous bone.^118^ Platelet concentrates (PCs), such as PRP or PRF, release growth factors (PDGF, TGF-β, IGF-1) that enhance tissue regeneration.^119^ In vitro, PRP stimulates MSC proliferation and suppresses inflammation.^120^ Clinically, PCs improve nonunion healing rates,^121^ with Ranjan et al. ^122^ observing successful union in 21/25 delayed fracture cases. Combined with BMA or biomaterials, PCs synergistically enhance repair,^123,124^ though long-term studies show inconsistent improvements over standard treatment.^125^ Nevertheless, research also suggests that different preparation and activation methods of PCs, as well as the composition of platelet dense granules, may have potential negative effects on bone healing.^126^

#### Biomaterials

Biomaterials are essential in bone regeneration by protecting cells from mechanical forces, enhancing cell retention, and providing structural support.^127^ Effective bone regeneration requires biomaterials need to exhibit three key features: osteoinduction, osteoconduction, and osteointegration. Polymers and ceramics are widely used in bone tissue engineering, each with distinct advantages and limitations.

Natural polymers, such as collagen, chitosan and alginate, are biologically active and biocompatible. Collagen I, a major extracellular matrix component, is osteoconductive^128^ but degrades rapidly, necessitating composites with bioceramics (e.g., hydroxyapatite (HA), tricalcium phosphate (TCP)) or growth factors.^129,130^ Collagen-based materials, including membranes, sponges, hydrogels, and scaffolds, have demonstrated success in bone defect repair and osteogenic differentiation.^131,132^ Chitosan, a derivative of chitin, modulates osteoblast and osteoclast activity^133^ and enhances drug delivery and tissue adhesion due to its cationic nature.^134^ However, its insufficient mechanical strength require combination with other biomaterials to enhance osteoconductivity.^135^ Alginate, derived from brown algae, has good gel-forming ability and biodegradability but lacks cell adhesion. Combining it with HA or chitosan enhances its mechanical strength and osteogenic potential.^136,137^

Synthetic polymers like polylactic acid (PLA), polyglycolic acid (PGA), poly(lactic-co-glycolic acid) (PLGA), and polycaprolactone (PCL) offer biocompatibility and tunable degradation.^138^ PLA’s properties depend on crystallinity and molecular weight, but its brittleness and acidic degradation byproducts limit its use.^139^ Blending PLA with bioceramics (e.g., TCP, magnesium) or polydopamine enhances osteoconductivity and mechanical stability.^140,141^ PLGA’s degradation rate and mechanical properties can be adjusted by varying lactic and glycolic acid ratios,^142,143^ but its hydrophobic nature and acidic degradation microenvironment hinder cell adhesion.^144^ Combining PLGA with nano-HA or akermanite (AKR) improves osteogenic differentiation and angiogenesis.^145^ PCL, known for its toughness, is ideal for 3D-printed bone implants but requires surface modifications (e.g., β-TCP coating, polydopamine/HA functionalization) to enhance cell adhesion and osteogenesis.^146^

Ceramics, particularly HA and TCP, are widely used due to their resemblance to bone’s inorganic composition.^147^ HA exhibits high osteoconductivity and osteoinductivity, promoting osteoblast adhesion and osseointegration,^148^ but its poor mechanical strength limits load-bearing applications.^149^ TCP, especially β-TCP, is more resorbable and osteoconductive, with optimized pore sizes (300–500 μm) enhancing cell differentiation and growth factor delivery. Combining HA and β-TCP forms biphasic calcium phosphate (BCP), which offers tunable bioactivity and degradation rates. Higher TCP content accelerates resorption and osteogenesis,^150^ making BCP scaffolds effective carriers for drugs and growth factors, thus enhancing bone regeneration.^151^

## Precise monitoring of bone healing microenvironment

While previous sections established that targeted bio-augmentation osteosynthesis can restore dysregulated bone healing toward physiological or accelerated repair through precise modulation of key phases, a critical gap persists in obtaining real-time feedback to guide such precision. This impedes optimization across multiple dimensions: (1) Anti-/pro-inflammatory cytokine dosing and timing remain unguided during inflammatory dysregulation due to lacking immediate assessment of local immunomodulatory efficacy; (2) Bioactive agent therapeutics (e.g., BMP-2) exhibit narrow therapeutic windows where insufficient dosing fails to augment healing while excess induces osteolysis; (3) Catastrophic infection complications demand instant detection of pathogen presence, identity, viability, and resistance; (4) Determining the quantity, subtypes, and corresponding protein expression of relevant cells within the bone healing microenvironment is essential for developing effective therapeutic strategies for bone healing; (5) Mechanostimulation requires temporal balance—early callus formation benefits from controlled mobility whereas late mineralization necessitates rigid fixation—yet current methods lack dynamic monitoring. Conventional non-invasive imaging (CT/MRI/X-ray/ultrasound) only evaluates macro-scale structural endpoints, failing to probe the local microenvironment, while invasive lab tests incur prohibitive delays (hours/days). Biosensor address these limitations by enabling rapid, specific, quantitative assessment at/near the injury site. Leveraging accessible biological samples (intraoperative specimens, drains, local injectates), biosenor facilitates real-time microenvironmental insight and immediate intervention adjustment to minimize complications and accelerate repair. This review focuses specifically on optical sensing strategies for monitoring bone healing microenvironments.

### Monitoring local inflammation in bone injury

During normal bone healing, levels of various inflammatory biochemical molecules are dynamically balanced. However, systemic or local variations can disrupt inflammatory homeostasis within the healing microenvironment, triggering reactive changes in biochemical levels. Notably, fluctuations between pro-inflammatory cytokines (e.g., TNF-α, IL-6) and anti-inflammatory cytokines (e.g., IL-4, IL-10) provide valuable information for assessing healing progress. Consequently, timely monitoring of these antagonistic cytokine fluctuations is crucial for guiding precise therapeutic interventions. To achieve this, researchers have developed various biosensing strategies. Wang et al. reported a phase-transition singularity-based 2D nanomaterial plasmonic biosensor achieving an ultralow detection limit (LOD) of 10⁻¹⁵ mol/L for TNF-α via enhanced plasmonic effects.^152^ Ryu et al. utilized liquid crystal orientation combined with detection/capture antibody pairs for TNF-α, achieving a linear range of 5.00–500 pg/mL with an LOD of 1.08 pg/mL and LOQ of 3.56 pg/mL.^153^ Cennamo et al. developed an IL-6 biosensor by combining self-assembled monolayers of identical antibodies with two distinct plasmonic probes, achieving femtomolar and picomolar LODs, validated in saliva and serum.^154^ Giorgi-Coll et al. employed aptamer-induced aggregation of AuNPs, causing a visible color change (red to pink), enabling rapid and highly sensitive IL-6 detection (LOD = 1.95 μg/mL) within 5 min.^155^ Wang et al. implemented label-free fiber-optic plasmonic resonance sensing for CRP detection. Monoclonal antibodies were immobilized via a dopamine crosslinker, yielding a linear response (R² = 0.97) from 0.01 μg/mL to 20 μg/mL.^156^ Baek et al. described an IL-10 detection method using colloidal gold nanoparticles. Binding to gold bars induced a red shift in the localized surface plasmon resonance extinction peak, achieving nanomolar-level detection.^157^ Building upon this foundation, our group previously enhanced the fluorescence of AuNCs by loading them onto ZIF-8 surfaces.^158^ This material was integrated into a hydrogel-based portable sensor for convenient monitoring. Further refinement involved Ag⁺ doping, synthesizing dual fluorescence-enhanced AuAg-ZIF. Combining this material with signal amplification and interpretable machine learning techniques enabled real-time, convenient, and accurate detection of key inflammatory cytokines (pro-inflammatory TNF-α, anti-inflammatory IL-10) and the inflammation marker CRP within the inflammatory steady state. This approach provides immediate feedback for precise clinical intervention in local inflammatory homeostasis during bone injury.^159^

### Monitoring bioactive factors

To enable rapid, sensitive, and simultaneous detection of multiple BMPs, Herrera et al. established a C2C12 cell line stably transfected with a reporter plasmid. This plasmid contained a BMP-responsive element (BRE) fused to the Id1 promoter and a luciferase reporter gene. Exposure of this cell line to different BMPs induced luciferase expression, quantified using a luminometer. This assay achieved picomolar-level detection of various BMPs within 48 h.^160^ Leveraging the ability of rhBMP-2 to significantly increase alkaline phosphatase (ALP) activity in C2C12 cells, Lian et al. measured ALP activity at 405 nm using a microplate reader as an indicator of rhBMP-2 bioactivity in vitro. Highly sensitive quantification was achieved by applying a four-parameter logistic model.^161^ For precise quantification of BMP-2 concentration immobilized on biomaterials, Wojciechowski et al. utilized base-catalyzed deprotection of Fmoc groups, generating dibenzofulvene as a mole ratio marker. UV-Vis spectroscopy accurately predicted the concentration of immobilized BMP-2 (R² = 0.96), determining the level required to stimulate osteogenesis in hMSCs.^162^

For VEGF detection, Li et al. constructed an aptasensor for VEGF detection by integrating DNA assembly with structural switching and isothermal amplification. The change in fluorescence intensity exhibited a good linear relationship with VEGF concentration over the range of 5 to 400 pg/mL, with a detection limit of 3.5 pg/mL (S/N = 3). The sensor was successfully validated in clinical serum samples.^163^ Leveraging the property of the cationic comb-type copolymer poly-L-lysine-graft-dextran (PLL-g-Dex) to accelerate DNA hybridization/strand exchange and stabilize DNA assemblies, Han et al. investigated its chaperone-like activity. This facilitated a G-quadruplex-based fluorescent DNA biosensor to achieve highly sensitive detection of vascular endothelial growth factor (VEGF), with a detection limit as low as 23 pmol/L.^164^ Deb et al. developed a fluorescent immunosensor based on a sandwich strategy utilizing carbon dot fluorescence. The sensor recorded the fluorescence intensity of the carbon dots in response to varying VEGF concentrations. This yielded a linear response over a wide range from 0.1 fg/mL to 10 pg/mL, with a detection limit of 5.65 pg/mL.^165^

For PDGF detection, Zhou et al. employed an aptamer-based machine utilizing a fluorescence-quenched, hairpin-structured aptamer probe. Binding of the target PDGF-BB to this probe induced structural and conformational changes in the aptamer, resulting in fluorescence restoration. This approach enabled highly sensitive detection of PDGF-BB, achieving a detection limit as low as 3.2 pmol/L.^166^ Building on aptamer recognition of PDGF-BB, Kan et al. integrated magnetic separation and fluorescence amplification modules in a plug-and-play fashion. This integration enabled highly sensitive detection of PDGF-BB, achieving a detection limit as low as 2.4 pmol/L.^167^ Yan et al. developed a sandwich-type chemiluminescent biosensor for PDGF-BB detection using magnetic beads as carriers. The chemiluminescent signal was generated by the reaction of 3,4,5-trimethoxyphenylglyoxal with guanine bases in the PDGF-BB aptamer strand. A good linear relationship was observed between chemiluminescence intensity and PDGF-BB concentration over the range of 4 ×10⁻¹⁰ to 2 ×10⁻⁸ mol/L, with a detection limit of 0.15 nmol/L.^168^

### Monitoring orthopedic infections

Assessment of host inflammatory response and pathogen detection constitute the dual pillars of orthopedic infection diagnosis.^169^ Diagnosis based on inflammation-related biomarkers in biological samples (e.g., blood, synovial fluid) is established in clinical guidelines, and corresponding devices have been developed for preliminary clinical use. However, the lack of point-of-care test (POCT) pathogen detection hinders precise intervention in bone healing during infection. This section provides a preliminary overview of POCT approaches for pathogen detection, covering four aspects: presence, species identification, viability, and antimicrobial resistance (AMR). Unlike diseases targeting predefined cellular features, the invading pathogens in orthopedic infections are typically unknown a priori. This complexity complicates detection using classical antibodies, aptamers, or nanobodies in vitro, limiting their clinical applicability. Pattern recognition receptors (PRRs) have garnered diagnostic interest due to their ability to recognize pathogen-associated molecular patterns (PAMPs) common to diverse bacterial species. Engineered chimeric human opsonin Fc-mannose binding lectin (FcMBL) conjugated to magnetic beads has demonstrated >80% capture efficiency for various Gram-positive bacteria, Gram-negative bacteria, and fungi. Leveraging structural differences among Gram-positive bacteria, Gram-negative bacteria, and fungi, Zhou et al. developed a microenvironment-sensitive aggregation-induced emission luminogen (AIEgen). This method enables rapid (<5–8 h) visual differentiation: Gram-negative bacteria (light pink), Gram-positive bacteria (orange-red), and fungi (bright yellow), facilitating clinical validation for urinary tract infections.^170^ He et al. established a dual-modal colorimetric-fluorescence method based on the differential permeability of the DNA-binding dye GelRed into bacteria with varying viability. Using *E. coli* O157:H7 as a model, this approach quantifies bacterial viability within 15 min, detecting as low as 0.1% dead bacteria.^171^ Zhao et al. integrated concanavalin A-modified gold nanoparticles (ConA-AuNPs), vancomycin-modified gold nanoparticles (Van-AuNPs), and polymyxin B-modified Prussian blue nanoparticles (PMB-PBNPs) onto a single platform. This method determines MIC within 4–8 h and has undergone clinical validation.^172^ Recently, Kim et al. reported an integrated pathogen identification and antimicrobial susceptibility testing (AST) platform in Nature (2024).^173^ Utilizing synthetic β-2-glycoprotein I peptides, this platform selectively recovers diverse microbial pathogens from whole blood for species identification and AST. It achieved concordance rates of 100% for species identification and 94.9% for AST compared to standard methods, significantly reducing the time to 13 ± 2.5 h.

### Others

Flow cytometry provides valuable insights into characterizing cell types and quantities. This technique successfully discriminates differences in lymphoid, hematopoietic, and endothelial progenitor cells across different bone marrow sources, thereby identifying optimal harvest sites for bone marrow aspiration.^174^ Furthermore, flow cytometry effectively monitors cell delivery efficiency, proliferation, and distribution patterns.^175–177^ Beyond cellular characteristics, variations in protein content revealed by proteomics offer new perspectives for analyzing bone healing. Grgurević et al. identified distinct plasma proteomic profiles among patients with differing bone healing outcomes using proteomic and metabolomic analyses.^178^ Disruptions in protein transport associated with these differences led to lipid metabolism dysregulation, ultimately impairing the bone healing process. Additionally, proteomic studies have identified variations in anti-hypoxia protein levels as a factor associated with the inhibition of bone regeneration.^179^

Real-time strain evaluation of implants is crucial for studying bone injury healing and postoperative prosthetic failure. Pelham et al. developed an in-situ strain sensor featuring a cantilevered indicator needle sensitive to plate bending. Displacement relative to an internal scale is visible on standard radiographs, with a 2.25 mm displacement observed in cadaveric experiments.^180^ However, high device variability and the requirement for radiographic imaging limit clinical utility. Klosterhoff et al. achieved real-time, on-demand monitoring of in vivo mechanical boundary conditions within bone defects using integrated strain sensors.^181^ Their fixator initially doubled deformation amplitude through load-sharing, subsequently nearly tripling mineralized bridging and increasing bone formation by >60%, effectively promoting bone healing. Hu et al. employed laser direct writing (LDW) technology to pattern conductive features (minimum sheet resistance <1.7 Ω sq⁻¹) onto carbon fiber-reinforced polyetheretherketone (CFR-PEEK) orthopedic implants.^182^ These features function as strain sensors. The fabricated sensor exhibited excellent linearity (R² = 0.997) within its operating range (0–2.5% strain). Integration of the sensor with a Bluetooth module paves the way for developing personalized smart orthopedic devices.

## Remaining challenges for improved clinical efficacy

The initial inflammatory phase is a cornerstone in the complex cascade of bone healing, where a precise and well-orchestrated release of cytokines and growth factors is essential for initiating the reparative processes. This phase is marked by the influx of immune cells and the formation of a provisional matrix that sets the foundation for subsequent tissue regeneration. The controlled delivery of bioactive molecules such as BMPs and TGF-β during this phase is paramount, as it influences cell recruitment, proliferation, and differentiation. Achieving spatiotemporal precision in vivo is challenging but crucial for optimizing healing outcomes. It requires advanced drug delivery systems that can respond to specific physiological cues or external triggers, ensuring that the bioactive molecules are delivered at the right location and at the right time. This level of control can potentially enhance the efficiency of bone regeneration, reducing healing times and improving the quality of repaired tissue.

### Detailed characterization of the local microenvironment

Following an injury, a ruptured blood vessel causes blood to flow into the injured area and a hematoma form.^3^ The fibrin clot in the blood acts as a temporary matrix that attracts immune cells such as macrophages and neutrophils.^183^ These cells initiate the inflammatory response by secreting cytokines (e.g., TNF-α, IL-1, and IL-6) that promote the recruitment of inflammatory cells to remove bone debris, fight infection, and promote the proliferation of endothelial cells and fibroblasts in preparation for the bone formation phase.^3^ Macrophages play a key role in the inflammatory microenvironment, and they can be polarized as M1 or M2. M1 macrophages promote the inflammatory response by secreting pro-inflammatory cytokines such as TNF-α and IL-1β, whereas M2 macrophages inhibit the inflammatory response and promote tissue repair by secreting anti-inflammatory cytokines such as IL-10 and TGF-β. In addition, the inhibitory state of inflammation or acute inflammatory storm is in turn due to its depletion of bone healing substances.

### Better control of the spatial and temporal doses and delivery of bioactive molecules in vivo

In the domain of bone healing, achieving precise control over the spatial and temporal dosing of bioactive molecules in vivo is essential for enhancing clinical outcomes. Advanced drug delivery systems that can respond to specific physiological or external cues are vital for the precise modulation of drug release.^184^ For instance, Lutolf et al. developed a PEG-based hydrogel that integrates MMP-sensitive peptide sequences to achieve an enzyme-triggered release of BMP-2, thereby promoting bone regeneration in a rat skull defect model.^185^ Additionally, Gan et al. utilized pH-sensitive mesoporous silica nanoparticles for the dual delivery of BMP-2 and dexamethasone, enhancing bone regeneration through pH-modulated drug release.^186^

When it comes to external stimulus-responsive drug delivery systems, near-infrared (NIR) light-triggered drug release systems demonstrate significant potential. Wang et al. employed PLGA scaffolds containing black phosphorus to achieve NIR light-triggered strontium release, which promoted bone regeneration in a rat femoral defect model.^187^ Moreover, an ultrasound-triggered drug release system was also utilized to control the release of BMP-2, fostering bone formation in a mouse model.^188^

Beyond the studies, numerous research teams have developed a variety of innovative drug delivery systems. Kim et al. leveraged an MMP-sensitive hydrogel for the controlled release of BMP-2, promoting the healing of cranial defects in rats,^189^ while Hsu et al. created a cathepsin K-sensitive PEG hydrogel for the precise control of drug release during bone resorption through enzyme-triggered drug release.^190^ To further improve clinical efficacy, researchers are exploring a range of stimulus-responsive materials, including heat-sensitive liposomes,^191^ photo-sensitive nanomaterials,^187^ and ultrasound-sensitive liposomes.^188^ These materials can alter drug release rates in response to external stimuli, providing new strategies for drug delivery during bone healing.

In the study of bone-targeted drug delivery systems, Reis et al. developed a thermo-responsive polyelectrolyte composite coating for controlled release of Bortezomib.^192^ Aw et al. achieved sequential release of multiple drugs using titanium dioxide nanotube arrays.^193^ Matsuo et al. investigated injectable magnetic liposomes as a novel carrier for BMP-2 delivery and promoted bone formation in a rat bone defect model.^194^ Although numerous studies have made significant progress, challenges remain in achieving clinical translation. Future studies need to further optimize these delivery systems to improve their biocompatibility, biodegradability, and to ensure precise and safe drug release. Additionally, large-scale clinical trials are a critical step in validating the effectiveness of these delivery systems.

## Challenges and perspectives

The intricate balance between inflammatory microenvironments and reparative substance availability remains central to bone healing, yet significant challenges hinder its precise modulation. A primary challenge lies in decoding the spatiotemporal dynamics of inflammatory signals and their interplay with repair mechanisms. Chronic inflammation, driven by aging or metabolic diseases, disrupts critical processes such as macrophage polarization and osteoprogenitor recruitment, while acute inflammatory dysregulation destabilizes provisional matrices and delays angiogenesis. Current strategies, including cytokine-targeted therapies or MSC delivery, often lack the precision to address phase-specific microenvironments, risking unintended suppression of essential repair pathways. Equally critical is the sustained replenishment of reparative resources within the evolving fracture niche. Systemic conditions exacerbate local nutrient deprivation and oxidative stress, impairing MSC differentiation and extracellular matrix mineralization. While biomaterials like calcium phosphate scaffolds aim to mimic the native microenvironment, their static designs struggle to adapt to the dynamic demands of healing stages. For example, early-phase scaffolds may inadvertently trap inflammatory debris, while late-phase materials could hinder remodeling by resisting osteoclastic activity. Future efforts should prioritize microenvironment-responsive systems, such as biomaterials releasing anti-inflammatory agents or nutrients in response to real-time biochemical cues. Advanced tools like single-cell sequencing could map microenvironmental heterogeneity across healing phases, enabling personalized interventions. Additionally, exploring systemic influences, such as the gut-bone axis or microbial metabolites, may uncover novel targets for modulating local inflammation. Ultimately, bridging the gap between microenvironmental insights and clinical translation requires interdisciplinary collaboration. By addressing these challenges, next-generation therapies could restore the delicate equilibrium between inflammation and repair, offering tailored solutions for patients with compromised bone healing.

## Conclusion

In conclusion, a precisely regulated inflammatory response and the availability of reparative substances are essential for effective bone healing. Chronic and excessive acute inflammation can hinder the healing process, while timely resolution of inflammation and replenishment of reparative substances are crucial for successful bone regeneration. Advances in biomaterial science offer innovative solutions to modulate the inflammatory microenvironment and address substance depletion, showing great potential for improving clinical outcomes in bone healing. Further research is necessary to optimize these approaches and translate them into effective clinical therapies.
